# Some Useful Analgesics

**Published:** 1903-04

**Authors:** J. Morgan Howe

**Affiliations:** New York


### SOME USEFUL ANALGESICS.

BY J. MORGAN HOWE, NEW YORK.

Wine of Opium.—This preparation has been recommended for odontalgia; for treating pulps and gingival tissues; but it has been of special service to me in relieving pain in pulpless teeth that continued to ache after local conditions had been made favorable and other applications had failed. Applied in the root-canal, it often soothes the irritated periapical tissues.

Mentho-phenol.[1]—Used in the same manner and for the same purpose as above mentioned; has been also a valuable remedy in these cases. Its decided analgesic properties may be proved also when applied to an aching pulp, or to painful tissues of a toothless socket after extraction. It is a remedy worth remembering, when a patient is suffering pain from either of these conditions. It has marked antiseptic properties also, but is not a disinfectant, in the degree required for the treatment of putrescent conditions.

Aristol.—This compound is soluble in ether, chloroform, and oils, and in mentho-phenol. It is a germicide and antiseptic, and also has decided analgesic properties. Dr. Jack reported, several years ago, that he used a saturated solution in gaultheria for a final dressing of root-canals in all cases.[2] A solution in mentho-phenol

[1] Made by melting together three parts menthol crystals with one part phenol crystals.—International Dental Journal, 1897, page 591.

[2] Ibid., 1897, page 371.

has proved effective in allaying pain. I am unable to say whether such solution is more efficacious than either ingredient alone, but there is no doubt that both have decided analgesic properties. I have proved this many times with regard to aristol, by using it, in an ethereal solution, in the root-canals of aching teeth. There is an objection, however, to the use of such a solution, in that the evaporation of the ether leaves the aristol in a gummy state, adhering to the instrument, and troublesome to manipulate. The color of aristol, and the fact that it contains more than forty-five per cent. of iodine, suggest caution in using it in anterior teeth, for fear of staining the dentine.

